# Multiparametric Optical Bioimaging Reveals the Fate of Epoxy Crosslinked Biomeshes in the Mouse Subcutaneous Implantation Model

**DOI:** 10.3389/fbioe.2020.00107

**Published:** 2020-02-19

**Authors:** Vadim Elagin, Daria Kuznetsova, Ekaterina Grebenik, Denis A. Zolotov, Leonid Istranov, Tatiana Zharikova, Elena Istranova, Anastasia Polozova, Dmitry Reunov, Alexandr Kurkov, Anatoly Shekhter, Elvira R. Gafarova, Victor Asadchikov, Sergey M. Borisov, Ruslan I. Dmitriev, Elena Zagaynova, Peter Timashev

**Affiliations:** ^1^Institute of Experimental Oncology and Biomedical Technologies, Privolzhsky Research Medical University, Nizhny Novgorod, Russia; ^2^Institute for Regenerative Medicine, Sechenov First Moscow State Medical University, Moscow, Russia; ^3^Shubnikov Institute of Crystallography, Federal Scientific Research Centre “Crystallography and Photonics” Russian Academy of Sciences, Moscow, Russia; ^4^Institute of Analytical Chemistry and Food Chemistry, Graz University of Technology, Graz, Austria; ^5^School of Biochemistry and Cell Biology, University College Cork, Cork, Ireland; ^6^Institute of Photonic Technologies, Federal Scientific Research Centre “Crystallography and Photonics” Russian Academy of Sciences, Moscow, Russia; ^7^Department of Polymers and Composites, N.N. Semenov Institute of Chemical Physics, Moscow, Russia

**Keywords:** decellularized tissue, biomeshes, bovine pericardium, epoxy crosslinking, hypoxia, *in vivo* biodegradation, optical bioimaging, PLIM

## Abstract

Biomeshes based on decellularized bovine pericardium (DBP) are widely used in reconstructive surgery due to their wide availability and the attractive biomechanical properties. However, their efficacy in clinical applications is often affected by the uncontrolled immunogenicity and proteolytic degradation. To address this issue, we present here *in vivo* multiparametric imaging analysis of epoxy crosslinked DBPs to reveal their fate after implantation. We first analyzed the structure of the crosslinked DBP using scanning electron microscopy and evaluated proteolytic stability and cytotoxicity. Next, using combination of fluorescence and hypoxia imaging, X-ray computed microtomography and histology techniques we studied the fate of DBPs after subcutaneous implantation in animals. Our approach revealed high resistance to biodegradation, gradual remodeling of a surrounding tissue forming the connective tissue capsule and calcification of crosslinked DBPs. These changes were concomitant to the development of hypoxia in the samples within 3 weeks after implantation and subsequent induction of angiogenesis and vascularization. Collectively, presented approach provides new insights on the transplantation of the epoxy crosslinked biomeshes, the risks associated with its applications in soft-tissue reconstruction and can be transferred to studies of other types of implants.

## Introduction

Biomeshes based on decellularized bovine pericardium (DBP) are widely used in reconstructive surgery by virtue of their ready availability and attractive biomechanical properties (Baharuddin et al., 2002; Colombo et al., 2009; Guerette et al., 2009; Limpert et al., 2009; Schlee et al., 2012; Mallis et al., 2017). They are already being applied to intracardiac surgery (Gabbay et al., 1984), abdominal wall repair (Limpert et al., 2009), treatment of gingival recession (Schlee et al., 2012), bone and periodontal defects (Stavropoulos et al., 2011), duraplastics (Baharuddin et al., 2002), ophthalmology (Gupta et al., 2002; Koay et al., 2008; Quaranta et al., 2013), the repair of rectovaginal septum defects (Guerette et al., 2009), as dental membranes and venous stent covers (Colombo et al., 2009). However, the xenogenic nature of the biomesh source prompts an immune response in the human body and facile biodegradation. Elimination of immunogenicity is usually achieved by means of decellularization and/or crosslinking. In early 1990s, epoxy compounds attracted a wide attention as a class of crosslinkers owing to their anti-calcification effect. Epoxy compounds are proved to prevent biodegradation and calcification of bioprosthetic materials while preserving their pliability and cytocompatibility (Imamura et al., 1989; Xi et al., 1992; Sung et al., 1993; Bre et al., 2014; Rezvova and Kudryavceva, 2018). However, these findings are valid for native, intrinsically immunogenic, tissues; while no comprehensive *in vivo* studies on acellular epoxy crosslinked biomeshes were performed. Currently, decellularization is a prerequisite preliminary step in the fabrication process for providing satisfactory integration of the xenomaterial into the patient’s tissue (Oswal et al., 2007; Dong et al., 2009; Mendoza-Novelo et al., 2011; Stavropoulos et al., 2011; Pagoulatou et al., 2012). The effects of the epoxy crosslinking on acellular biomeshes have been comprehensively investigated *in vitro* (Perme et al., 2009; Grebenik et al., 2019), while the data on the structural alterations undergoing upon implantation are incomplete and often are based on the use of very diverse experimental assessment methodologies.

Currently, dominating methods for analysis of biomeshes’ behavior in animal model are histology and direct mechanical measurements. Potentially, employment of novel cutting-edge techniques will enable to obtain frequently missing information on calcification dynamics, vascularization and hypoxia and other factors-dependent tissue remodeling. Thus, fluorescence stereomicroscopy permits to assess a biodegradation rate based on fluorescence signal intensity. Alterations in a collagen fiber structure can be studied by a non-linear microscopy techniques. X-ray computed microtomography provides information about interaction between biomeshes and animal body. Studying oxygenation and metabolism is highly important for tracking vascularization.

In present work, we have performed a complex *in vitro/in vivo* imaging study of the process of implant integration into living tissue using promising biomesh based on an epoxy compound, ethylene glycol diglycidyl ether (EGDE) crosslinked DBP. Our approach is based on a set of advanced techniques including fluorescence stereo microscopy, X-ray computed microtomography, multiphoton tomography based on two-photon excitation fluorescence (TPEF), and second harmonic generation (SHG), and phosphorescent nanosensor-mediated O_2_ (hypoxia) imaging. This is a first instance for combining such highly informative multi-parametric analysis of the transplantation efficiency and subcutaneous integration of the epoxy crosslinked biomeshes. Altogether, this approach is attractive for the characterization of the material behavior with *in vivo* models and is useful for planning and adequate clinical treatment.

## Materials and Methods

### Biomesh Preparation

Bovine pericardia were obtained directly from a local slaughterhouse and transported to our laboratory in a cold hypertonic solution. The pericardia were carefully cleaned of excess fat and connective tissue. The decellularization was achieved by treatment of the tissue with a mixture of 1M sodium hydroxide and 0.85M sodium sulfate for 2 h at 20°C followed by rinsing with water and neutralization with 4% (wt/v) boric acid for 1 h. The decellularized tissues were then rinsed with water thrice for 30 min, under continuous shaking. Decellularization success was assessed with the aid of the nuclear stain, ethidium homodimer (EthD-1), using a Nikon A1 MP confocal microscopy system (Nikon Instruments) with a 561 nm laser (Em: 570–620 nm). Decellularized tissues were then crosslinked in a phosphate buffer (pH 9) containing 5% (wt/v) of EGDE – a protocol adopted from Grebenik et al. (2019). Crosslinked samples were extensively washed, lyophilized and then sterilized with γ-radiation.

### Scanning Electron Microscopy

For SEM study, 1 cm^2^ sample patches or transverse 23 μm-thick microtome sections were prepared. Prior to sectioning, the biomeshes were dehydrated in ethanol and embedded in paraffin. The sections were mounted on standard microscopy slides, deparaffinized, plated with gold in a Magnetron Sputtering System LEYBOLD LH Z400 (Germany) and examined with a Zeiss Supra 40VP scanning electron microscope (Carl Zeiss Group, Germany) using an acceleration voltage of 10 kV and a working distance of 4.3 mm.

### MTT-Test

An MTT [3-(4,5-dimethylthiazol-2-yl)-2,5-diphenyltetrazolium bromide] extraction test was adopted from ISO 10993. Sample extracts were prepared as follows: tissue specimens (three 1 cm^2^ pieces) were finely minced and incubated in 1 ml of culture medium for 24 h at 37°C. Serial dilutions of the intact (non-crosslinked) and epoxy crosslinked samples in DMEM/F12 culture medium supplemented with 100 U/mL streptomycin, 100 g/mL penicillin, 1% (v/v) GlutaMAX (Gibco), 5% (v/v) fetal bovine serum (HyClone); with positive control [sodium dodecyl sulfate (SDS)] and negative control (culture medium alone) in triplicates (100 μl) were added to a subconfluent monolayer of L929 murine fibroblast cells in 96-well plates. The plates were then incubated for a further 24 h at 37°C in 5% (v/v) CO_2_ in air before the effects of the extracts on cell viability were determined using the MTT-test. For the MTT-test, the extract and control media were replaced with 100 μL of the MTT solution (0.5 mg/mL in medium without supplements) and were incubated in a CO_2_-incubator at 37°C for 3 h. After discarding the MTT solution, 100 μL aliquots of dimethyl sulfoxide were added to all the wells and the plates swayed. The color developed was quantified by measuring absorbance at 550 nm (reference 650 nm) using a microplate photometer *(Multiskan FC*, Thermo scientific).

### *In vitro* Biodegradation Assay

The susceptibility to collagenase digestion was studied in Collagenase A (from *Clostridium histolyticum*) solution. Approximately, 4 mg (dry weight in triplicates) of each sample were weighed. To the weighed samples, 0.2 ml aliquots of 2.5 mg/ml Collagenase A solution in Tris buffer (50 mM, pH 7.4) containing 10 mM calcium chloride and 0.02 mg/ml sodium azide (Paneco, Russia) were added. The samples were incubated at 37°C for 24 h. Then, the samples were centrifuged at 12,000 rpm for 10 min (using an A-14, Thermo Electron Corporation, Jouan-type centrifuge), washed with phosphate buffered saline (PBS), and the remaining tissue samples then dried in an oven at 60°C for 20 h. Finally, they were weighed and the weight loss was calculated by a paired comparison of before and after the treatments. The biodegradation in papain was studied in a solution of papain (Karipazim, Georgia, 100 U/ml) prepared in Tris buffer (50 mM, pH 8) containing 10 mM cysteine, 20 mM ethylenediaminetetraacetic acid (EDTA) and 0.02% sodium azide (Paneco, Russia) after 6 days, followed by the same measurement protocol.

### Analysis of Biomeshes Degradation in Animal Model

All *in vivo* experiments were approved by the Ethics committee of the Privolzhsky Research Medical University (Nizhny Novgorod, Russia). We performed *in vivo* experiments on 30 male BALB/c mice (the mean weight body 20 g). The animals were divided into two equal groups. The animals were anesthetized with Zoletil (80 mg kg^–1^) and fixed on a support holder. The mouse back hairs were shaved and the underlying skin cleaned and sterilized using chlorhexidine gluconate. Throughout the surgical procedures all strict sterility measures were upheld for survival surgeries. 7-mm incisions were made on the dorsal section of each mouse to implant the intact and epoxy crosslinked DBP samples. The biomeshes were separately and independently implanted into each mouse (2 samples of DBP of the same type). The incisions were sutured and medical glue was topically applied to the surgery sites to prevent infection. 10 samples of each DBP type were harvested and analyzed with following techniques at each time point (3, 6, and 12 weeks) after the implantation.

### Fluorescence Stereomicroscopy

The main components of the DBP samples are collagen and elastin fibers. Collagen and elastin have several fluorescent moieties in the ultraviolet and visible spectral regions (Croce and Bottiroli, 2014) that may account for the DBP fluorescence. To assess biodegradation of the biomeshes, we used a special technique developed earlier for bone implants having a fluorescence signal (Timashev et al., 2016; Kuznetsova et al., 2017). In brief, we analyzed the fluorescence of implanted pericardium tissue using an Axio Zoom V16 (Carl Zeiss, Germany) fluorescence stereomicroscope and ImageJ 1.43u software (National Institutes of Health, United States). In the fluorescence images, the areas of all the samples were identified and the ‘Integrated density’ (IntDen) parameter was measured. The area of the biomeshes was evaluated with the ‘Area’ parameter. In addition, we made light images of both the intact and the crosslinked samples to visualize growing blood vessels.

### Multiphoton Tomography Analysis

A multiphoton tomograph (MPTflex^®^, JenLab, Germany) was used to image the TPEF and the SHG of the DBP samples. The images were acquired through a 40×, 1.3 NA oil immersion objective. The excitation wavelength was set at 750 nm for acquiring the both TPEF and SHG signals of collagen. Backward-directed SHG signals were detected in the 373–387 nm range. A 409–660 nm emission filter was used for TPEF acquisition. The acquisition time of an XY image (512 × 512 pixels and 220 × 220 μm) was approximately 15 s. TPEF and SHG images were taken through the samples from the surface at z-steps of 10 μm. At least 5 z-stacks were acquired for each sample. For each image in the acquired stacks the average values of the photon intensities for TPEF and SHG were calculated by manually selecting 30 × 30 pixel zones as regions of interest.

### X-Ray Computed Microtomography

The explants were dehydrated in ethanol, embedded in paraffin and analyzed using a custom-built X-ray microtomograph as described in Buzmakov et al. (2018). This microtomograph was equipped with a laboratory X-ray source GE ISOVOLT 3003 with a tube having Cu anode (energy E_Ka__1_ = 8 keV) anode. The measurements were performed in monochromatic radiation using a pyrolityc graphite crystal C (0001) (monochromatization *dE/E* = 0.1). The total 400 projections were obtained, each 0.5° apart, and recorded by CCD-detector with 9 × 9 μm pixel size. The exposure time for each projection was 5 s. The 3D images were acquired by a modified algebraic method using CGLS regularization (Buzmakov et al., 2019).

### Histological and Immunohistochemical Analysis of Harvested Samples

For histological, immunohistochemical and confocal microscopy analysis, tissue samples with implanted intact DBP and EGDE biomeshes were fixed in 10% neutral buffered formalin. The samples were dehydrated and embedded in paraffin. Ten sections from the middle of each paraffin block were prepared with a Leica SM 2000 microtome (Germany). Transverse serial 4 μm thick sections were stained with hematoxylin and eosin and van Gieson’s picrofuchsin to analyze the general histoarchitecture of the tissues and collagen structures. Von Kossa staining was applied to assess the degree of calcification.

In immunohistochemical study, endothelial cells of blood vessels were visualized with rabbit monoclonal antibodies to CD31 (ab182981, Abcam, United States) at 1:2000 dilution. Goat anti-rabbit IgG cross-adsorbed antibody conjugated with Alexa Fluor 488 (A-11008, Thermo Fisher Scientific, United States) was applied as secondary antibody at 1:1000 dilution. Immunohistochemical study with primary mouse monoclonal antibodies to CD 68 (KP1 clone, ÌÀ5-13324, Thermo Fisher Scientific, United States) at 1:100 dilution and biotin-goat anti-mouse IgG (H + L) secondary antibodies (Life Technologies Corporation, United States) at 1:500 dilution was performed to visualize macrophage and giant cell reaction.

We investigated the histological and immunohistochemical samples by light and fluorescent microscopy using a LEICA DM4000 B LED microscope equipped with a LEICA DFC7000 T digital video camera and LAS V4.8 software (Leica Microsystems, Germany).

NucBlue^TM^ Fixed Cell Reagent (Invitrogen) was used for staining cell nuclei. The tissue sections were deparaffinized and stained for 30 min at 37°C according to the manufacturer instructions followed by thorough rinsing with PBS. Fluoromount^TM^ Aqueous Mounting Medium (Sigma) was applied to the tissue sections then covered with a coverslip and sealed. For confocal imaging, we used the LSM 880 Airyscan (Carl Zeiss, Jena, Germany) scanning laser confocal microscope.

The morphometric study of tissue reaction to scaffold implantation was performed using the microscopic images taken at the magnification of × 630 in 10 representative fields of view (FOV). We analyzed the numbers of cells invading the scaffolds and in scaffold capsules, the capsule thickness, the degree of vascularization of the scaffolds and their capsules. The degree of maturation of scaffold capsules and the degree of calcification and resorption of scaffold material were also assessed by a semi-quantitative evaluation (Supplementary Tables S1, S2).

### Nanosensor-Mediated O_2_ Imaging

Conjugated polymer-based cationic SI-0.2^+^ and zwitter-ionic SI-0.1^+^/0.1^–^ and SI-0.05^+^/0.15^–^ were synthesized and handled as described previously (Dmitriev et al., 2015). Briefly, nanosensors respond to changes in phosphorescence intensity and lifetime with increasing at low O_2_ (hypoxia) and decreasing at high O_2_. These changes can be calibrated and used for quantification (Papkovsky and Dmitriev, 2018). Due to their highly charged surface groups, nanosensors can stick and remain bound to various protein-based scaffold materials. For biomeshes staining, non- and EGDE-crosslinked DBP scaffolds were incubated with different SI nanosensors (100 μg/ml, 1 h, 37°C) in PBS, subsequently washed with PBS (1–2 times) and used for *in vitro* and *in vivo* experiments.

For *in vitro* evaluation of stability, SI-0.1^+^/0.1^–^ stained scaffolds were incubated in PBS supplemented with 10% fetal bovine serum (Sigma) over 0–14 days period at 37°C and measured on a custom-made phosphorescence lifetime imaging (PLIM) microscope as described previously (Dmitriev et al., 2013). Briefly, samples were imaged using 10x/0.3 Neofluar objective (Zeiss) using 390 nm LED and emission collected with 635 nm long pass filter. The acquired images were exported as RGB TIFF files and measured (with background correction) in ImageJ (Fiji.sc) software.

For *in vivo* evaluation SI-0.1^+^/0.1^–^ – stained samples were implanted subcutaneously to Balb/c female mice. Each animal was bearing both intact DBP and EGDE samples. Sensor phosphorescence was measured on explanted samples after 1, 2 and 3 weeks, using confocal scanning system for macroscopic objects DCS-120 MACRO (Becker & Hickl GmbH, Germany). Phosphorescence was excited by 375 nm diode laser with 60 ps pulse width and emission collected with 610 nm long pass filter. The phosphorescence signal was collected for 60 s. The acquired images had 256 × 256 pixels in resolution and 1024 time channels. The field of view was 10 m × 10 mm. The calculation of phosphorescence lifetime was done in SPCImage (Becker & Hickl GmbH, Germany) using the mono-exponential fitting as described previously (Dmitriev et al., 2015).

### Statistical Analysis

The mean values (M) and standard deviations (σ) were calculated for all the data. A one-way ANOVA with Fisher’s *post hoc* test and a Student’s *t*-test were used to compare the data (*p* ≤ 0.05 was considered statistically significant).

The morphometry study results were statistically analyzed using a standard program package GraphPad Prism version 8.2 (GraphPad Software, San Diego, CA, United States) and Statistica version 12.0 (StatSoft, Inc., Tulsa, OK, United States). Anderson–Darling test, D’Agostino & Pearson test, and Shapiro–Wilk test used for testing the normality of distribution of data. Then the geometric mean was accounted for a parameter “the capsule thickness” in 10 field of view in each tissue sample, for other parameter median were performed. The normally distributed data was accounted using two-way ANOVA followed by Sidak’s multiple compassion test. Other data was accounted with compassions in Mann–Whitney *U*-test. The differences between the studied groups was considered statistically significant at *p*-value less than 0.05.

## Results

### Sample Preparation and Characterization

The bovine pericardium tissue was successfully decellularized using alkaline treatment as was indicated by staining the samples with EthD-1 before and after decellularization. Confocal laser scanning microscopy revealed numerous stained cell nuclei present on the smooth side of the native pericardium tissue, whereas there were almost no cell nuclei on the decellularized sample (Supplementary Figure S1).

**FIGURE 1:**
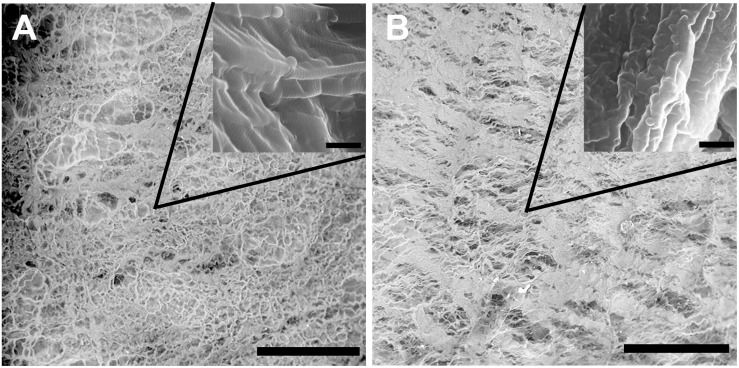
Scanning electron microscopy characterization of the intact **(A)** and epoxy crosslinked DBP **(B)**. Scale bar is 200 μm. The inserts are the same sample at higher magnification, scale bar is 500 nm.

Scanning electron microscopy was performed to assess the structural properties of the intact and epoxy crosslinked DBPs. The SEM analysis showed that the micro- and ultrastructural properties of the intact DBP and EGDE samples were different (Figure 1). The intact DBP had a compact unbroken arrangement of fibers with axial periodicities (D-period) of the collagen molecules. Crosslinking with EGDE affected the axial periodicity and changed the surface pattern, increasing the roughness. The average pore size was 10–140 μm in intact DBP and 10–130 μm – after epoxy crosslinking. However, the shape of the pores shifted from oval to more elongate with the tendency to fiber tightening and parallel packing. The fibril thickness increased upon cross-linking from 111.9 ± 20.8 to 117.1 ± 11.2 nm.

### *In vitro* Assessment of Biocompatibility of the Intact and Epoxy Crosslinked Biomeshes

**FIGURE 2:**
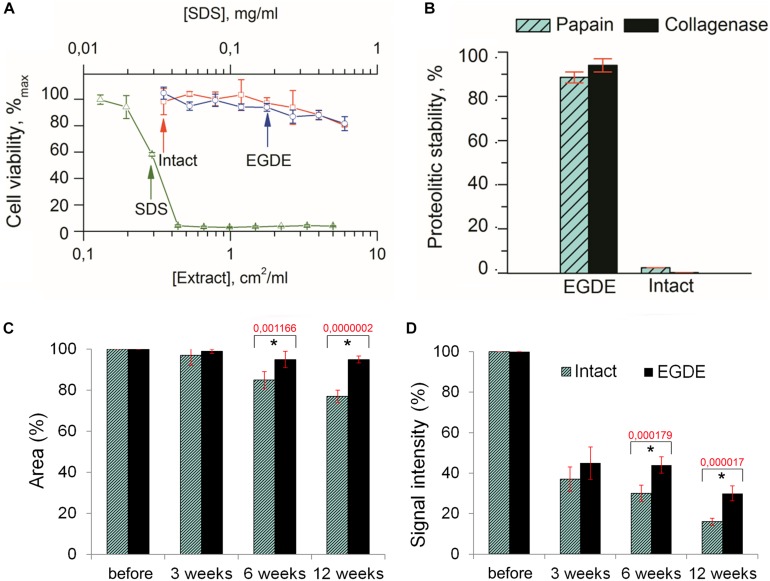
Biodegradation analysis of the pericardium tissue *in vitro* **(A**,**B)** and *in vivo* **(C**,**D)**. **(A)** Cytotoxicity assays of an intact and of an EGDE biomeshes with murine fibroblasts L929 (SDS was used as a positive control); **(B)** biodegradation of the intact DBP and EGDE samples in Collagenase and Papain solutions; **(C)** quantitative analysis of the fluorescence signal intensity; **(D)** quantitative analysis of the implants area. *N* = 10, data shown as mean ± SD. *Statistically significant differences are presented between the intact and the EGDE samples, *p*-values are shown.

To evaluate the biocompatibility of the biomeshes we carried out MTT enzymatic reduction assays. The metabolic activity of actively respiring L929 murine fibroblast cells was found not to be influenced by the presence of EGDE crosslinks (Figure 2A). EGDE did not increase the cytotoxicity level of the biomeshes, and the relative cell viability was above 70% for the highest concentration of the sample extracts. SDS was used as a positive control, causing a remarkable level of cell death (IC_50_ ∼ 0.03 mg/ml).

Analysis of the biodegradation of the DBP samples in Collagenase and Papain solutions revealed that the EGDE biomeshes possessed a level of proteolytic stability several orders higher than that of the intact DBP (Figure 2B).

### Analysis of Biomeshes Degradation in Animal Model

Both intact and epoxy crosslinked DBP samples were found to have strong fluorescence signals. The integral value of the signal intensity of the samples depends on the fiber structure and the total amount of protein in the pericardium tissue. The loss of fluorescence signal with time *in vivo* is proportional to the decrease in size of the DBP as it is degraded. Measurement of the signal intensity over time can therefore provide a relative assessment of DBP biodegradation. Collagen type I being a constituent of DBP is known to have strong SHG signal. The overall value of the mean signal intensity of the samples depends on the fiber structure and its packing.

Before implantation the intact and EGDE samples could be visualized as square-shaped structures with clear borders on stereomicroscopy (Supplementary Figure S2). The area and the fluorescence signal intensity (IntDen) were considered as 100%. Three weeks later, the borders of the samples still retained their shape, with the sample area still being about 97–99% of its initial value for all samples (Figure 2C), while the IntDen signal level had dropped by around two to three times (37% for the intact DBP and 45% for the EGDE sample; Figure 2D). Six weeks after the implantation the intact and epoxy crosslinked DBPs showed no further significant changes as compared with those at the 3-week time point. The areas were 85 and 95% for the intact and EGDE biomeshes respectively. The intact DBP had a more pronounced tendency toward a decrease in area. The IntDen signal level was 30% for the intact DBP and 44% for the EGDE sample. By week 12, the intact DBP area was 77% of its initial value, and its signal level had decreased to 16%. The EGDE biomeshes presented a more stable structure. Their areas and IntDen signals showed almost no change as compared to those at 3 and 6 weeks.

To evaluate the structure-functional alterations of DBPs during biodegradation we performed analysis of the shape of fibers as well as of the TPEF and SHG signal intensities by multiphoton tomography. The intact DBP samples were presented by dense collagen fibers with random direction (Supplementary Figure S3). The entangled collagen fibers had generally a cord-like shape and a tape-shape in a less. It was detected that the EGDE biomeshes consisted of dense regular collagen fibers were parallel to each other. By week 12 the intact DBP samples consisted of randomly oriented tape-shaped collagen fibers. No cord-like fibers were observed in the samples. In contrast, collagen fibers in the epoxy crosslinked DBPs did not change either shape or orientation. Analysis of intensities either TPEF or SHG revealed that both DBP samples had equal levels of each before implantation. However, biodegradation of the intact DBP led to a decrease of the TPEF value relative to that of the SHG, whereas, in the EGDE samples, the TPEF intensity dominated (Supplementary Figure S3).

**FIGURE 3:**
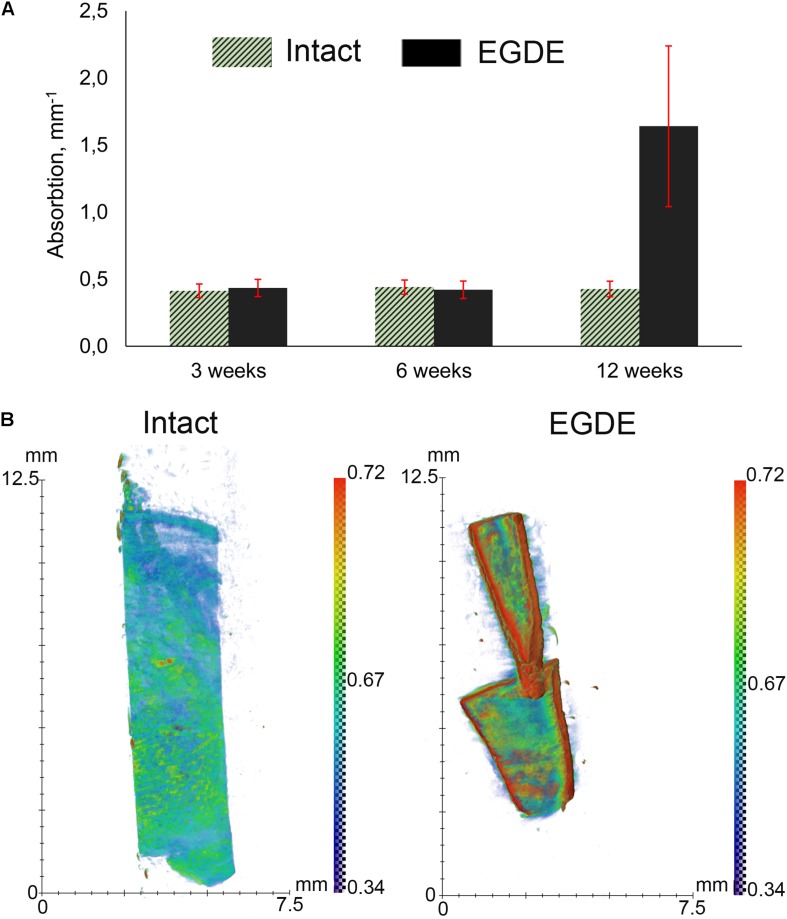
X-ray computed microtomography analysis of DBP samples: **(A)** average absorption coefficients of the samples at 3, 6, and 12 weeks after subcutaneous implantation; **(B)** X-ray absorption histogram of the samples after 12 weeks. The data show a threefold increase in the X-ray absorption by the EGDE samples at 12 week after implantation. *N* = 10, data shown as mean ± SD.

X-ray computed microtomography was used to analyze macrostructural alterations of biomeshes. The average values of the linear absorption coefficient were calculated for both DBPs samples at 3^rd^, 6^th^, and 12^th^ week after implantation (Figure 3A). The intact DBPs demonstrated the stable value over the experimental period. In contrast, the absorption coefficient of EGDE biomeshes dramatically increased at 12 week after implantation. For each sample, the values higher than 0.3 mm^–1^ were selected to build the distribution histogram (Figure 3B).

### Interaction of the Intact and Epoxy Crosslinked Biomeshes With Recipient’s Body

**FIGURE 4:**
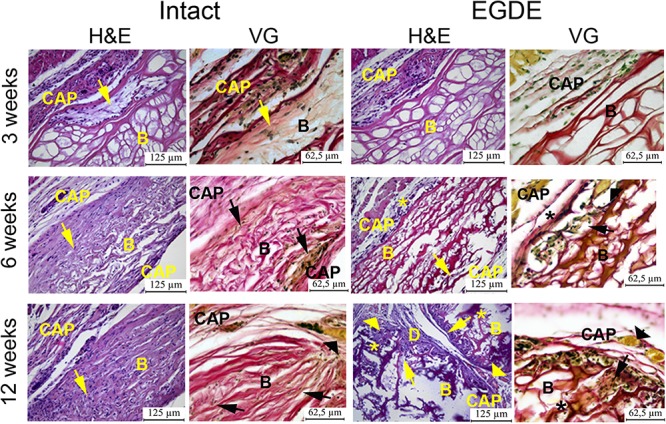
Histological analysis of intact DBP and EGDE biomeshes 3, 6, and 12 weeks after the implantation. Implanted DBP biomeshes (B) were surrounded by a capsule (CAP) consisting of mature granulation tissue (3 weeks) or mature connective tissue (6 and 12 weeks), which grew into pores forming connective tissue septa (arrows) containing blood vessels. Calcification (*) and defects (D) of some parts of the implants’ material were determined. Hyperemia (arrowheads) of the periphery of scaffolds. Scale bar for hematoxylin and eosin (H&E) staining is 125 μm, for van Gieson (VG) staining is 65.5 μm.

**FIGURE 5:**
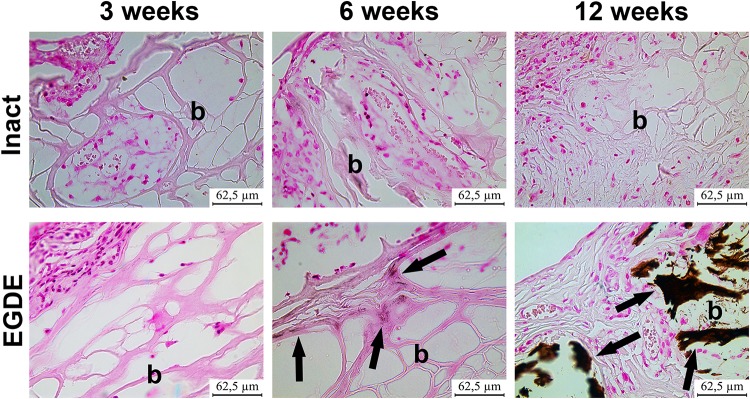
Analysis of calcification of intact DBP and EGDE biomeshes (b) 3, 6, and 12 weeks after the implantation: EGDE with intensive calcification (arrows). The von Kossa staining, scale bar is 62.5 μm.

**FIGURE 6:**
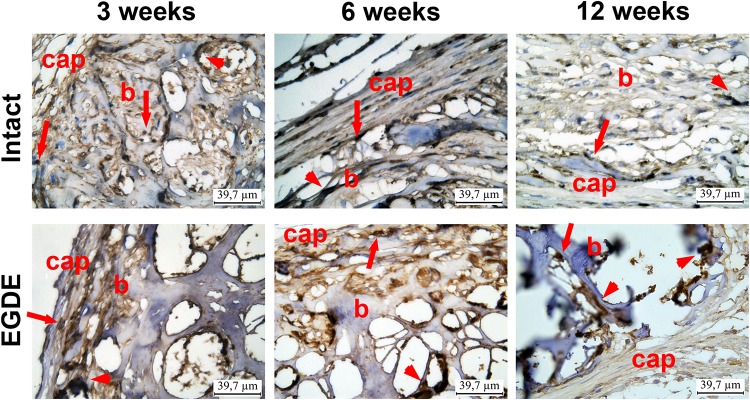
Immunohistohistochemical analysis of intact DBP and EGDE biomeshes 3, 6, and 12 weeks after the implantation. CD 68 expression in macrophages (arrows) and giant cells (arrowheads) in biomeshes (b) and in their capsules (cap). Scale bar is 39.7 μm.

Morphological and morphometric analyses (Figures 4–6 and Supplementary Tables S1, S2) and confocal microscopy analysis (Supplementary Figure S4) were performed in order to explain the changes in the intensity of fluorescence, SHG signal and X-ray absorption. 3 weeks after the operation, mature granulation tissue surrounded biomeshes of both DBP types forming a relatively thin capsules (Figure 4). Sponge shaped biomeshes had eosinophilic and fuchsinophilic septa. The capsules consisted of fibroblasts, sporadic small-caliber blood vessels, macrophages, foreign body giant cells, lymphocytes, and rare neutrophils. The capsules around intact DBP biomeshes were more mature than around epoxy crosslinked DBPs (Figure 4). However, the differences were statistically insignificant (Supplementary Table S3). They formed numerous septa in the peripheral pores of the biomeshes with rare small capillaries. In the EGDE samples, these septa were visually absent. The macrophage and fibroblast invasion into the intact DBP implant was more pronounced than that in the EGDE group (Figures 4, 5 and Supplementary Figure S4, Table S3). 6 weeks after the implantation, intact DBP biomesh material was severely resorbed by numerous macrophages inside and outside the scaffolds (Supplementary Table S3) and rare giant foreign body cells were present (Figure 6); the capsule was mature. Its connective tissue septa occupied most of the superficial pores and some deep pores of the biomeshes (Figure 4). The septa were very thin and consisted of immature connective tissue with an extremely scarce number of vessels and fibroblasts. In the epoxy crosslinked DBPs, the capsules had a similar structure, but it was noted to have a more prominent hyperemia, vascularization, and thickness (Supplementary Table S3). Moreover, rare connective tissue septa were observed only in the surficial pores and contained a significant number of giant cells (Figures 4, 6). The number of giant cells in both scaffold and surrounding capsule were similar to that in intact DBP group (Supplementary Table S3). Unlike the intact DBP samples, peripheral septa in this group were modestly calcified (Figures 4, 5), yet statistically insignificant. 12 weeks after the implantation of intact DBP biomeshes, structure of the capsules and connective tissue septa did not significantly differ from the previous period. However, connective tissue septa occupied almost all pores (Figure 4 and Supplementary Figure S4), the number of fibroblasts insight and outside the scaffolds decreased. The resorption was statistically different from that of EGDE group (Supplementary Table S3). In the EGDE biomeshes, capsules, and connective tissue septa structures did not differ much from the previous time point as well. However, the EGDE biomesh material underwent an intensive calcification increasing tissue fragility and resulting in large defects that occurred after implantation during the life of the animal (Figures 4, 5 and Supplementary Table S3). The defects were filled with septa consisting of immature connective tissue with a weak inflammatory infiltration and a prominent hyperemia. At the same time, the number of fibroblasts in the scaffolds and surrounding capsule decreased, and the capsule became thinner that in the intact DBP group (Supplementary Table S3). In spite of the prominent hyperemia in EDGE group, the total number of blood vessels outside the scaffold decreased compared to the previous time point and was comparable with that in intact DBP group (Supplementary Table S3).

**FIGURE 7:**
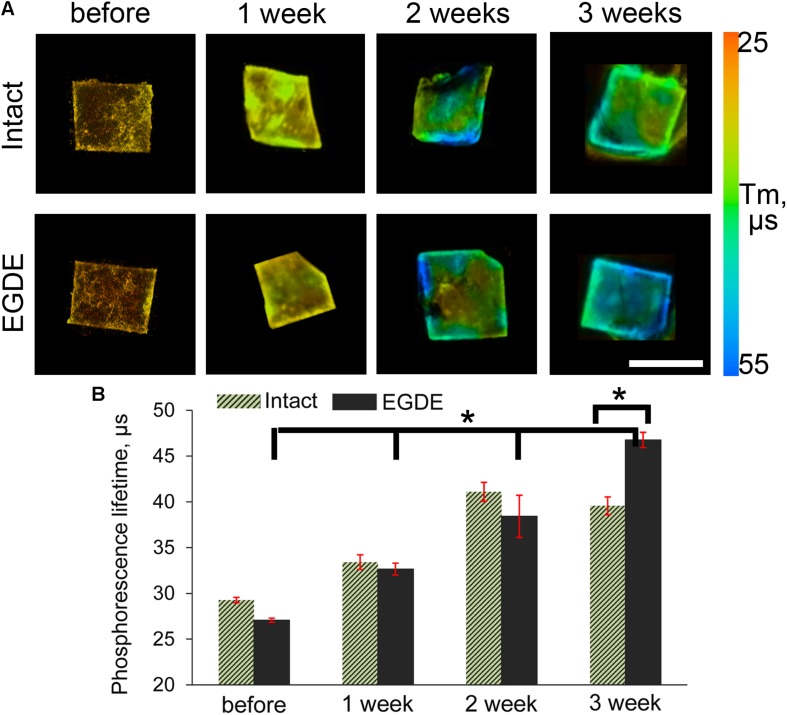
PLIM imaging of intact DBP and EGDE biomeshes: **(A)** hypoxia progress in DBP biomeshes implanted subcutaneously to Balb/c mice; **(B)** quantification of the mean phosphorescence lifetime (τm, μs) of O_2_-sensitive nanoparticles. Scale bar is 5 mm. *N* = 3, data shown as mean ± SD. *Statistically significant differences, *p* < 0.001.

We looked if the capsule formation could affect hypoxia in the implanted biomeshes. To test this, we used PLIM microscopy with the help of O_2_-sensitive nanosensors. Recent success in design of O_2_-and pH- sensing ‘hybrid’ tissue engineering materials (Jenkins et al., 2015; Yazgan et al., 2017; O’Donnell et al., 2018; Roussakis et al., 2019; Schilling et al., 2019) prompted us to stain the pericardial biomeshes with O_2_-sensitive polymer nanoparticles. We hypothesized that charged nanoparticles could experience hydrophobic interactions and provide strong non-specific adsorption with the crosslinker or the polypeptide chains of the DBPs. We evaluated a promising cationic and zwitter-ionic (mixed charge) SI nanosensors with ratiometric and two-photon excited blue/red emission (Dmitriev et al., 2015) for staining of the scaffolds (Supplementary Figure S5). The brightest signals were observed for zwitter-ionic SI-0.1^+^/0.1^–^ nanoparticles, which were chosen for subsequent *in vivo* experiments. We further evaluated the stability of the staining by incubating them in serum-containing PBS over the 2 weeks time at 37°C and found negligible decrease in the fluorescence of the stained scaffolds over the tested period (Supplementary Figure S6). Interestingly, both intact and epoxy crosslinked DBPs displayed stability of the staining with O_2_-sensitive nanoparticles. *In vivo* study showed that the mean values of phosphorescence lifetime of the sensor increased during the experiment, indicating decrease of oxygenation levels (Figures 7A,B). After 3 weeks the τ values of the EDGE samples grew in two times compared to those before implantation and reached nearly 0% O_2_ values (∼55 μs).

**FIGURE 8:**
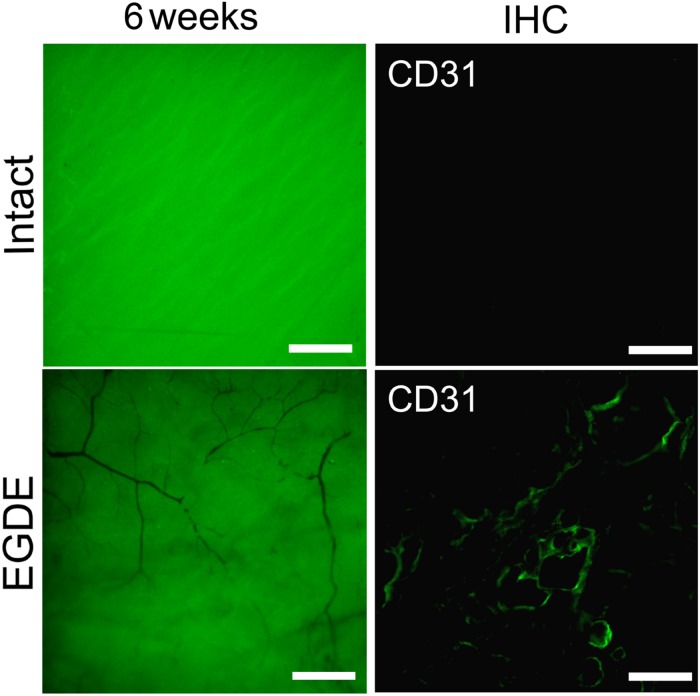
Estimation of the DBP vascularization: fluorescence bioimaging (fluorescence stereo microscopy, the scale bar is 500 μm); CD31 immunohistochemically (IHC) staining of a DBP cross-section 12-weeks after implantation (fluorescence microscopy, the scale bar is 100 μm).

A vascular network formation was observed in the capsule around the EGDE samples 6 weeks after the implantation. Vascular networks could be seen both at the borders and in the centers of all the EGDE samples by fluorescence stereo microscopy (Figure 8). By contrast, the capsule around the intact DBP samples were absolutely free from vascularization. The content of the blood vessels was assessed by CD31 immunohistochemical staining.

## Discussion

Biomeshes constitute frequently used collagen-based matrix derived from biological tissue through a decellularization process. Grafted biomeshes act as a regenerative framework that supports remodeling and new collagen deposition (Bielli et al., 2018). The effectiveness of transplantation depends on the ability of the biomeshes to integrate into the host tissue promoting the *de novo* formation of tissues similar to the normal ones. The post-implantation fate of biomeshes is therefore crucial for biomaterial selection, determining the long-term success of a tissue-engineered graft (Sung et al., 2004).

A great number of approaches are aimed to understand and improve the tissue remodeling of biomeshes (Sun et al., 2013). Since the crosslinking of the decellularized biomaterials impacts on both biodegradation and tissue regeneration (Bielli et al., 2018), stabilization with new crosslinkers and elimination of immunogenicity are highly important (Xu and Wang, 2012; Ma et al., 2014; Rothamel et al., 2014; Wang et al., 2016). The influence of these parameters on the regeneration of the tissues needs to be comprehensively investigated before such biomaterials can be appropriately selected for biomedical applications. There have been many studies in which the effects of decellularization and crosslinking have been evaluated *in vitro* (Pagoulatou et al., 2012; Xu and Wang, 2012; Bielli et al., 2018). Importantly, the rates of biomesh biodegradation can differ drastically between the *in vitro* and *in vivo* settings. However, only a small number of studies controlled the biodegradation rate of pericardium tissue after implantation into animals (Rothamel et al., 2005, 2014; Gardin et al., 2015). Unfortunately, most of these studies were based on histological and morphometric analysis of harvested implants.

In this work we took advantage of various *state-of-the-art* techniques helping to understand biodegradation of biomeshes: non-invasive fluorescence imaging developed for *in vivo* estimation of the dynamics of the biodegradation rate of such implants (Timashev et al., 2016; Kuznetsova et al., 2017), *in vivo* imaging of implant (de)oxygenation using O_2_-PLIM, *ex vivo* using multiphoton tomography, and conventional histological techniques. Before the implantation, we analyzed proteolytic stability and cytotoxicity of DBP samples *in vitro* (Figure 2). Our results demonstrated cytocompatibility of all DBP samples. We found that proteolytic stability of the intact and EGDE biomeshes showed different tendencies and rates of biodegradation and higher stability for EGDE samples. In a good agreement with literature, that epoxy crosslinking provides appropriate proteolytic stability but has no substantial effect on cytotoxicity, unlike that typical of traditional glutaraldehyde treatment (Sung et al., 1999; Delgado et al., 2015). However, we demonstrated that the differences in the proteolytic resistance of the intact and of the epoxy crosslinked DBPs were less profound in the experiments *in vivo* compared with the data from corresponding tests *in vitro* (Ma et al., 2014). Fluorescence technique revealed a reduction in the area of only 20% in the case of intact DBP samples (Figure 2C and Supplementary Figure S2). Meanwhile the signal intensities of both fluorescence and SHG decreased about five times (Figure 2D and Supplementary Figure S3). Histological analysis revealed that the both DBP samples were surrounded by capsule consisted of connective tissue. The capsule leads to attenuation of signal intensities and increase the light scattering effects. Moreover, inflammatory cells and fibroblasts presented in capsule provided remodeling collagen fiber. Changes in collagen structure were found in both DBP samples (Supplementary Figure S3). The next reason of signal intensities reduction is the significant calcification of the EGDE biomeshes revealed by histological analysis and X-ray microtomography. The calcification also increases fragility, which is likely to cause alteration of the surrounding tissues and supports the inflammatory reaction with severe angiomatosis and hyperemia (up to 12 weeks after implantation). Although earlier *in vitro* studies showed no effect of epoxy compounds on biomesh calcification while incubation in calcium phosphate solution (Vasudev and Chandy, 1997) or with cells grown on its surface (Connolly et al., 2005). Calcification of implants is a negative effect violating the main function of an implant. Currently patients with breast implants face the such problem (Toscani et al., 2013). Polymer medical devices used in cardiology are also subject to calcification (Schlieper et al., 2008). Characteristics of the implant surface considerably influence the calcification process, either due to its chemical composition (Kim et al., 2007) or due to its roughness (Wang et al., 2004; Grebenik et al., 2018). Surface chemistry influences the calcification process by complexation of chemical groups on the material surface with calcium or phosphorus ions leading to the mineralization. Calcification of the epoxy crosslinked DBP implants may be induced by several reasons. Firstly, using scanning electron microscopy, we have established a change in the topology of DBP as a result of epoxy crosslinking with the formation of roughnesses. Secondly, the appearance of inflammatory cells, first of all, macrophages, into the implantation area was established. It was earlier shown, that macrophages are colocalized with calcification areas for the atherosclerotic changes and the inflammation degree correlates with the calcification degree (Aikawa et al., 2007). Thirdly, increased vascularization of the peripheral parts of the implant and impaired outflow from the deep layers can provoke the accumulation of calcium salts from the blood. Probably, calcification-capable implants may be used to regenerate bone tissue, but this requires a careful future study.

Vascularization of the peripheral parts of the EGDE implant is supported by CD31 immunohistochemical staining that revealed endothelial cells migration. According to published data, the endothelial cells migration may be caused by chemoattractants, contained in large quantities in the extracellular matrix. Several studies have shown that the biodegradation products of the extracellular matrix of the pigs’ small intestine are capable to induce angiogenesis, mitogenesis, and cell differentiation (Hodde et al., 2001; Reing et al., 2009). However, in our work it was shown that the EGDE-treated DBP practically did not change in size during 90 days of biodegradation. Therefore, the chemoattractant release was prohibited compared to the intact sample. According to some studies, the calcification may trigger angiogenesis. *In vitro* study showed that calcification of extracellular matrix stimulates the synthesis and release of endothelial cell stimulating angiogenesis factor (ESAF) (Brown et al., 1994). It was also shown that scaffolds based on hydroxyapatite and dentin are able to stimulate angiogenesis during subcutaneous implantation in mice (Rucker et al., 2008). Thus, the calcification is the most appropriate mechanism of endothelial cell stimulation.

We also presented an innovative approach to understand implant oxygenation by labeling it with O_2_-sensing phosphorescent probe (Figure 7). While in general the methodology of *in vivo* O_2_ imaging is not new, until recently it was mostly focused on the use of vasculature- and interstitial space- specific probes (Esipova et al., 2019). Labeling scaffold materials with O_2_ probe having long retention time opens new prospective area in the implant-based imaging. Even though it was not achieved here, hypoxia imaging of surrounding scaffold area can represent exciting future venue for research. Thus, PLIM imaging confirmed strong implant deoxygenation during the first 3 weeks, which explains angiomatosis along the periphery of the epoxy crosslinked DBPs. It is known that angiogenesis is a component of chronic inflammation: many cells are capable of producing angiogenic factors when their environment becomes hypoxic or inflammatory (Jackson et al., 1997). In the case with EGDE sample, hypoxic conditions could be the main factor promoting the vasculogenesis and very interesting for future studies. The following decrease in the number of blood vessels and fibroblasts and the capsule thinning along with the prominent hyperemia are related to the connective tissue reorganization in chronic hypoxia and inflammation.

For the first time, we observed the coincidence of calcification and peripheral angiogenesis of the epoxy crosslinked DBPs, associated with hypoxic conditions inside the biomesh. In our work, we report on angiomatosis along the periphery of the epoxy crosslinked DBPs. This appeared in the remodeled tissue and no angiogenesis was detected in the implant itself. The hypoxia was likely caused by the increased proteolytic stability and calcification of the biomesh prohibiting and slowing down the tissue remodeling. *In vitro* biodegradation of the EGDE samples was much slower compared to the intact DBP both, in collagenase and papain solutions. In the work of Umashankar et al. (2012) glutaraldehyde-crosslinked DPB exhibited similar angiogenesis pattern in the periphery of the implant, chronic inflammation observed and related calcification.

Some works have shown that bone grafts containing a graded structure of bone tissue and a branching structure of vessel networks can promote both osteogenesis and angiogenesis (Serbo and Gerecht, 2013; Mercado-Pagan et al., 2015). Understanding of this phenomenon is critical for the design of new therapeutic approaches in soft tissue engineering.

In conclusion, we have carried out a complex *in vivo* study of the effects of epoxy crosslinking on the process of implant integration into living tissue. Structural studies were performed with highly informative and precise techniques including scanning electron microscopy, fluorescence stereo microscopy, X-ray computed microtomography, multiphoton tomography combined with TPEF and SHG. Epoxy crosslinked DBPs presented more stable structures. However, the differences in the proteolytic resistance of the intact and epoxy crosslinked DBP samples was less marked in the experiments *in vivo*. Using fluorescence bioimaging we have estimated vascularization of the implanted DBPs. We observed, for the first time, to the best of our knowledge, the promotion of biomesh vascularization with epoxy compound crosslinking associated with prohibited remodeling of the host tissue and hypoxia. The analysis of TPEF and SHG intensities revealed structural changes of the biomeshes after inoculation into a living organism. Severe calcification of EGDE samples was detected at 12^th^ week after subcutaneous implantation using histological analysis and X-ray computed microtomography. These results illustrate the effect of epoxy crosslinking on the development of DBP calcification in living organism. This unfavorable result should stimulate detailed studies such biomaterials. Presented multimodal imaging approach allows estimating the safety and long-term outcome of the biomesh used for soft tissue reconstruction.

## Data Availability Statement

All datasets generated for this study are included in the article/Supplementary Material.

## Ethics Statement

The animal study was reviewed and approved by Ethics committee of the Privolzhsky Research Medical University.

## Author Contributions

VE, DK, and EG: study concept and design. VE, DK, EG, DZ, AP, AK, ERG, and RD: data acquisition and analysis. LI, TZ, EI, DR, AS, VA, and SB: quality control of the data. VE, DK, EG, AK, and RD: manuscript preparation. EZ and PT: manuscript review. All authors read and approved the submitted version.

## Conflict of Interest

The authors declare that the research was conducted in the absence of any commercial or financial relationships that could be construed as a potential conflict of interest.
