# Supplementary material for: Multiparametric Optical Bioimaging Reveals the Fate of Epoxy Crosslinked Biomeshes in the Mouse Subcutaneous Implantation Model
Source: Front Bioeng Biotechnol. 2020 Feb 19;8:107. doi: 10.3389/fbioe.2020.00107 (PMC7042178; doi:10.3389/fbioe.2020.00107)
Supplement: Supplementary file 1 [file Data_Sheet_1.docx]

Supplementary Material

**
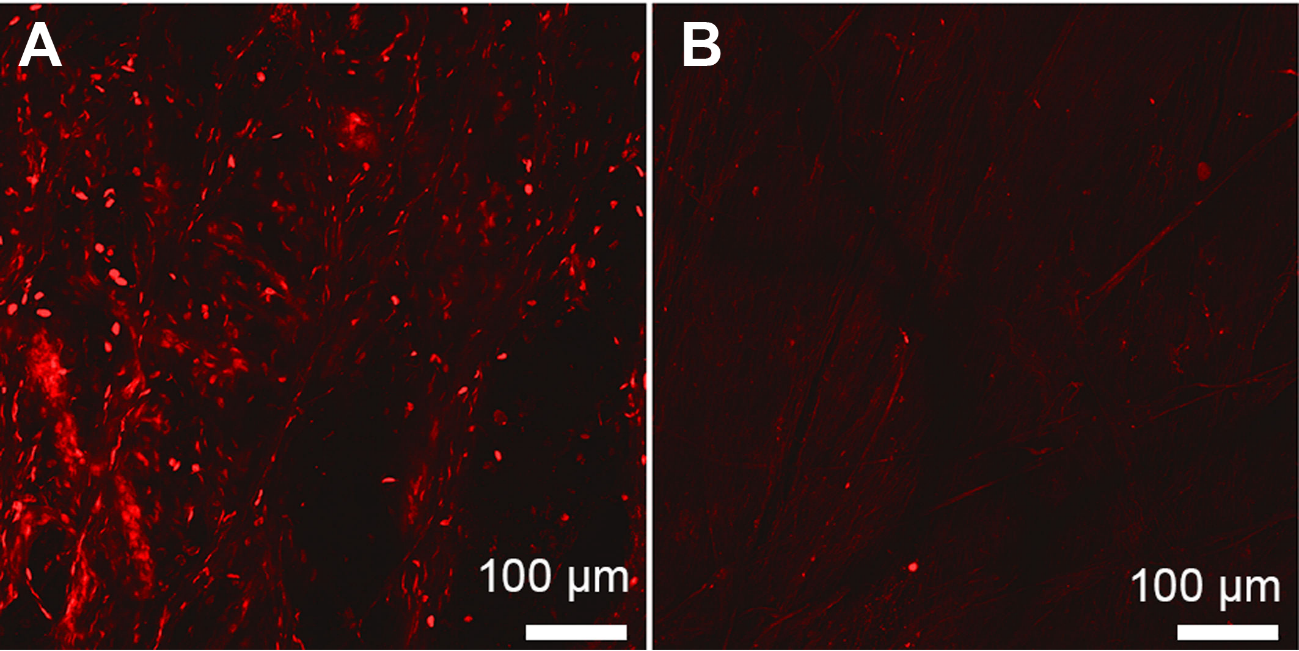
**

**Supplementary Figure S 1** Characterization of prepared bovine pericardium tissue. (A) decellularization assessment via ethidium homodimer-assisted confocal laser scanning microscopy. The removal of cell nuclei from the samples is confirmed by the lack of an ethidium homodimer signal at the smooth side of the DBP. Scale bar is 100 µm.


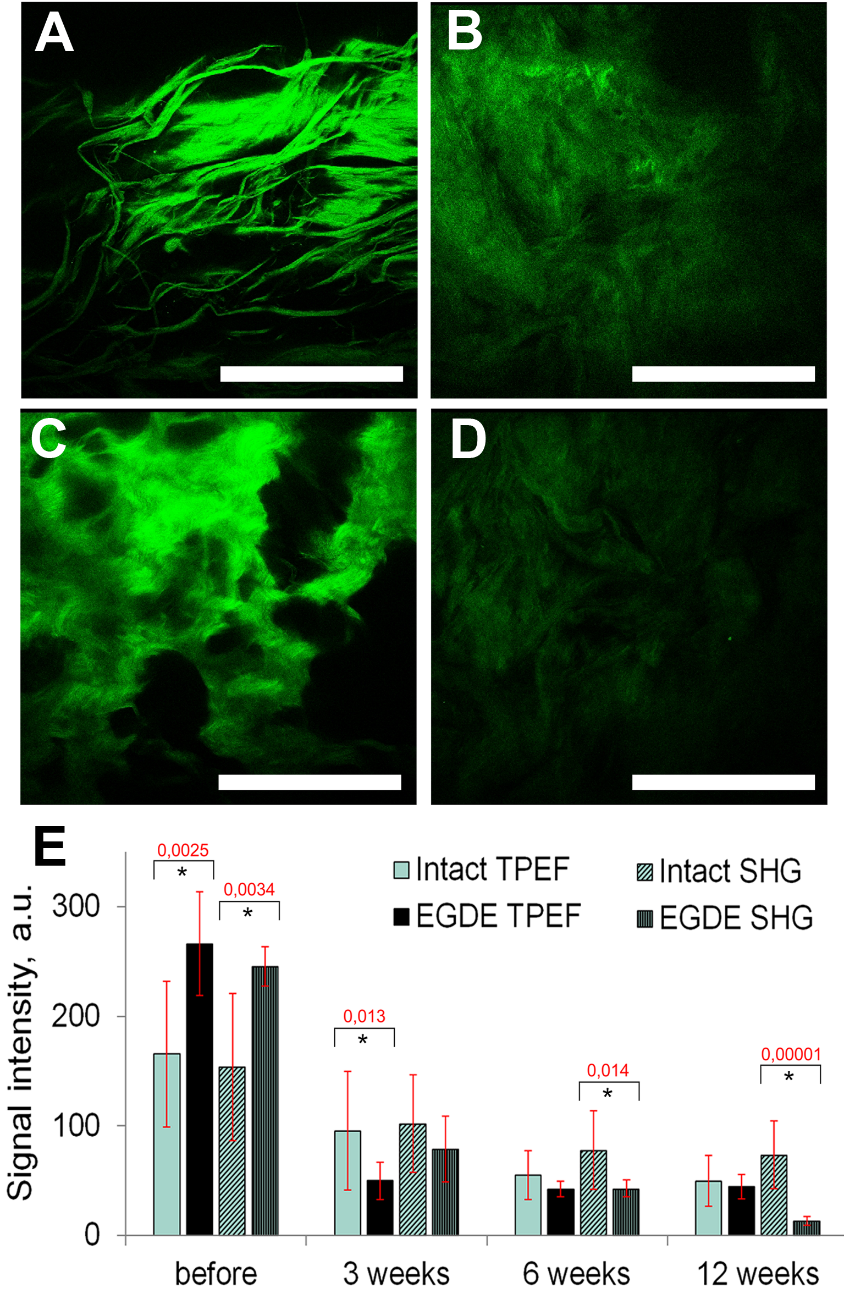


**Supplementary Figure S 2** SHG-based evaluation of the intact DBP (A&B) and EGDE (C&D) biomeshes structure before implantation (A&C) and after 12 weeks (B&D). Changing of the DBP biomeshes during biodegradation: analysis of intensities of SHG and TPEF (E). The scale bar is 100 µm. N=10 data shown as mean±SD * - statistically significant differences are presented between the intact and the EGDE biomeshes, p-values are shown.


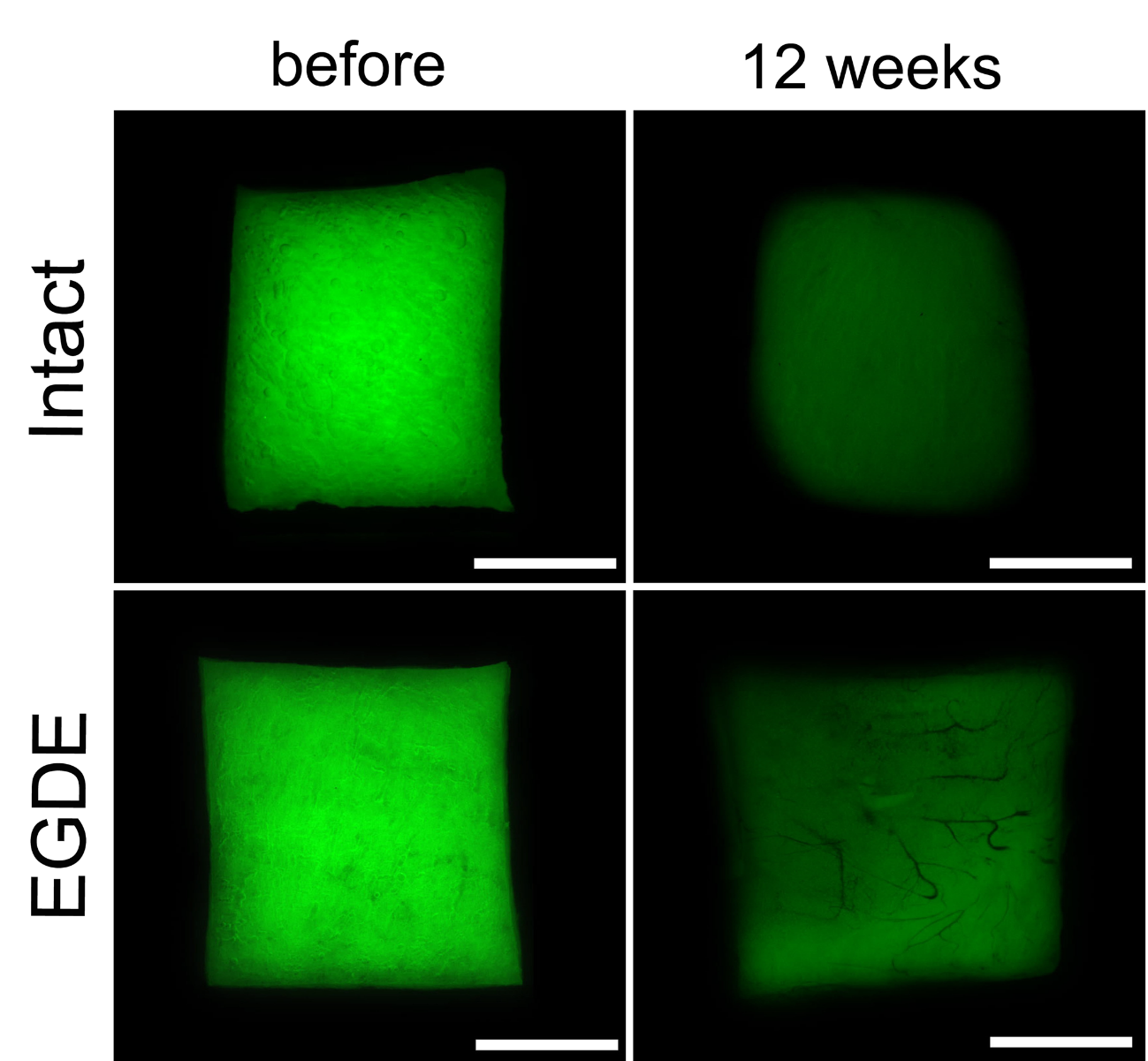


**Supplementary Figure S 3** Biomesh biodegradation analysis: fluorescence of the intact DBP and EGDE biomeshes before and 12 weeks after implantation into mice (fluorescence stereo microscopy, the scale bar is 2 mm).


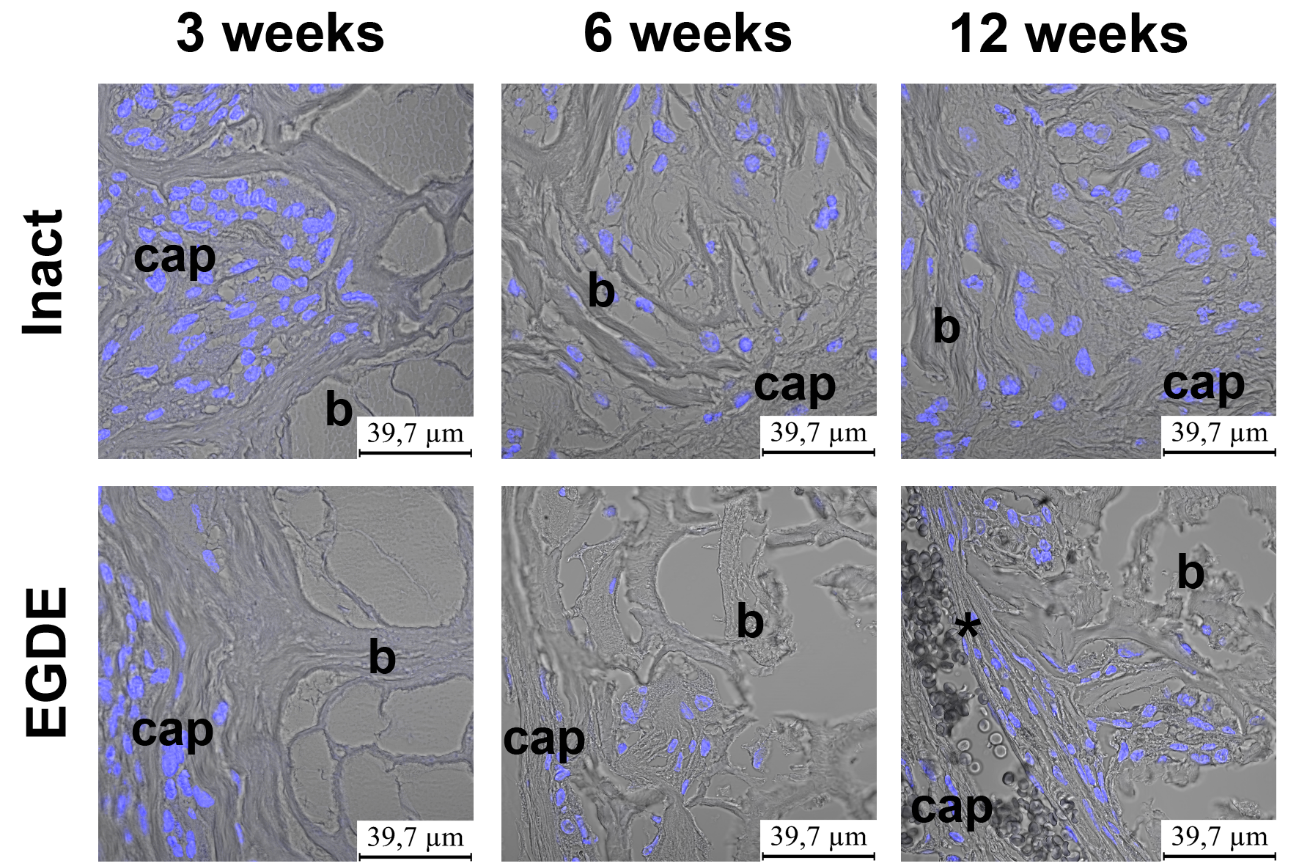


**Supplementary Figure S 4** Confocal imaging of the cells invading the intact DBP and EGDE biomeshes (b) and in their capsules (cap). 3, 6 and 12 weeks after the implantation; hyperemia (*) of the periphery of EGDE biomeshes at 12 weeks after the implantation. Scale bar is 39.7 μm.


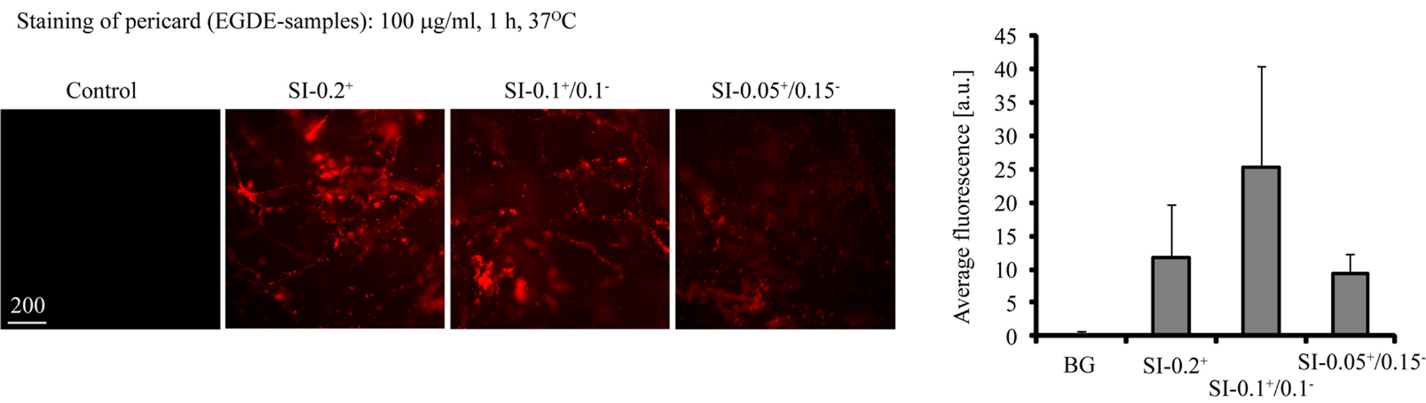


**Supplementary Figure S 5** Staining of the EGDE-crosslinked biomeshes with O_2_-sensitive nanosensors SI. Samples were incubated with different SI nanoparticles in PBS and measured by fluorescence microscopy (exc. 390 nm, em. 660 nm, control – unstained sample). Average background-corrected fluorescence intensities are shown on the right. Scale bar is in μm. N=3.


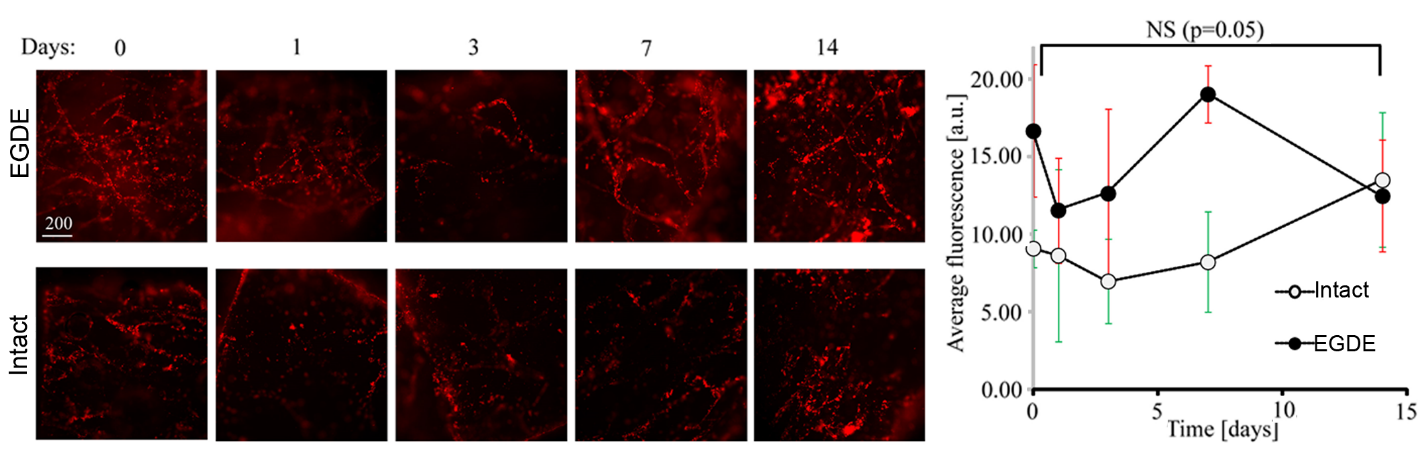


**Supplementary Figure S 6** Evaluation of SI-0.1^+^/0.1^-^ -stained biomeshes stability *in vitro*. Biomeshes were incubated in PBS / 10% FBS and measured by fluorescence microscopy (exc. 390 nm, em. 660 nm). Scale bar is in μm. N=3.

**Supplementary Table S1. A histological semiquantitative scoring system for the evaluation of a maturity of connective tissue capsules around scaffolds (Bardakova et al., 2019).**

| Points | Characteristics of a Capsule Around the Scaffold in each FOV |
| --- | --- |
| 0 | The capsule in all areas is immature (represented exclusively by granulation tissue) or mild focal fibrosis of granulation tissue in less than 25% of the capsule area |
| 1 | Mild focal fibrosis of granulation tissue in more than 25% of the capsule area |
| 2 | Pronounced focal or weak diffuse fibrosis of granulation tissue over 25% of the capsule area |
| 3 | Pronounced diffuse fibrosis of granulation tissue in more than 25% of the capsule area |

**Supplementary Table S2. A histological semiquantitative scoring system for the evaluation of scaffold lysis and calcification (Bardakova et al., 2019).**

| Points | Changes in a scaffold (scaffold lysis / calcification) in each FOV |
| --- | --- |
| 0 | No change or weak focal changes in less than 25% of the scaffold area |
| 1 | Weak focal changes in more than 25% of the scaffold area |
| 2 | Pronounced focal or weak diffuse changes in more than 25% of the scaffold  Area |
| 3 | Pronounced diffuse changes in more than 25% of the scaffold area |

**Supplementary Table S3. Summary table of morphometric analysis results**

| **Parameter (Median with 25th and 75th percentile, Mean±SD*) in 10 FOV** | **Method** | **3^rd^ week** | | | **6^th^ week** | | | **12^th^ week** | | |
| --- | --- | --- | --- | --- | --- | --- | --- | --- | --- | --- |
|  |  | **Intact DBP** | **EDGE** | **p-value** | **Intact DBP** | **EDGE** | **p-value** | **Intact DBP** | **EDGE** | **p-value** |
| Number of granulocytes inside the scaffold | Mann-Whitney U test | 0 | 0 | 1,000000 | 0 | 0 | 1,000000 | 0 | 0 | 1,000000 |
| Number of granulocytes in the scaffold capsule | Mann-Whitney U test | 0 | 0 | 1,000000 | 0 | 0 | 1,000000 | 0 | 0 | 1,000000 |
| Number of macrophages inside the scaffold | Mann-Whitney U test | 2- (from 2 to 3) | 0 | 0,000183** | 2 (from 1 to 2) | 0 | 0,000183** | 0 | 0 | 1,000000 |
| Number of macrophages in the scaffold capsule | Mann-Whitney U test | 9 (from 8 to 11) | 10 (from 9 to 11) | 0,623177 | 1 (from 1 to 1) | 0 | 0,000183** | 0 | 0 | 0,733730 |
| Number of giant cells inside the scaffold | Mann-Whitney U test | 0 | 0 | 0,969850 | 0 | 0 | 0,969850 | 0 | 0 | 0,969850 |
| Number of giant cells in the scaffold capsule | Mann-Whitney U test | 0 | 0 | 0,969850 | 0 | 0 | 0,969850 | 0 | 0 | 0,969850 |
| Number of lymphocytes inside the scaffold | Mann-Whitney U test | 0 | 0 | 0,969850 | 0 | 0 | 0,969850 | 0 | 0 | 0,969850 |
| Number of lymphocytes in the scaffold capsule | Mann-Whitney U test | 0 | 0 | 0,969850 | 0 | 0 | 0,969850 | 0 | 0 | 0,969850 |
| Number of plasma cells inside the scaffold | Mann-Whitney U test | 0 | 0 | 0,969850 | 0 | 0 | 0,969850 | 0 | 0 | 0,969850 |
| Number of plasma cells in the scaffold capsule | Mann-Whitney U test | 0 | 0 | 0,969850 | 0 | 0 | 0,969850 | 0 | 0 | 0,969850 |
| Number of fibroblasts inside the scaffold | Mann-Whitney U test | 4 (from 3 to 5) | 0 | 0,000183** | 23 (from 22 to 25) | 0 | 0,000183** | 16.5 (from 12 to 18) | 0 | 0,000183** |
| Number of fibroblasts in the scaffold capsule | Mann-Whitney U test | 21 (from 19 to 22) | 20 (from 19 to 21) | 0,449692 | 18.5 (from 18 to 19) | 20.5 (from 18 to 24) | 0,161973 | 4.5 (from 4 to 7) | 8 (from 7 to 9) | 0,015565** |
| Number of blood vessels inside the scaffold | Mann-Whitney U test | 0 | 0 | 0,969850 | 0 | 0 | 0,733730 | 0 | 0 | 0,969850 |
| Number of blood vessels in the scaffold capsule | Mann-Whitney U test | 0 | 0 | 0,733730 | 0 | 1 (from 0 to 1) | 0,025749** | 0 | 0 | 0,969850 |
| Capsule thickness around the implanted scaffolds, µm* | 2-way ANOVA foloved by Sidak's multiple comparisons test | 90.4±1.713 | 90.1±1.66 | 0,9995 | 29.5±4.53 | 64.1±15.51 | <0.0001** | 19.4±2.91 | 11.5±1.35 | 0,0356** |
| Degree of capsule maturation (score: 0-3) | Mann-Whitney U test | 1 (from 1 to 1) | 1 (from 1 to 1) | 0,969850 | 3 | 3 (from 2 to 3) | 0,273037 | 3 | 3 | 0,969850 |
| Degree of calcification of scaffold material (score: 0-3) | Mann-Whitney U test | 0 | 0 | 0,969850 | 0 | 0 | 0,969850 | 0 | 3 | 0,000183** |
| Degree of resorption of scaffold material (score: 0-3) | Mann-Whitney U test | 0.5 (from 0 to 1) | 0 | 0,064023 | 2 | 0 | 0,000183** | 2 | 0 | 0,000183** |

** Significant difference

Bardakova, K.N., Akopova, T.A., Kurkov, A.V., Goncharuk, G.P., Butnaru, D.V., Burdukovskii, V.F., et al. (2019). From Aggregates to Porous Three-Dimensional Scaffolds through a Mechanochemical Approach to Design Photosensitive Chitosan Derivatives. *Marine drugs* 17(1)**,** 48. doi: 10.3390/md17010048.
